# Feline Troglostrongylosis: Current Epizootiology, Clinical Features, and Therapeutic Options

**DOI:** 10.3389/fvets.2018.00126

**Published:** 2018-06-20

**Authors:** Paolo E. Crisi, Angela Di Cesare, Andrea Boari

**Affiliations:** Faculty of Veterinary Medicine, Veterinary Teaching Hospital, University of Teramo, Teramo, Italy

**Keywords:** troglostrongylosis, *Troglostrongylus brevior*, lungworms, kitten, wildcat

## Abstract

Parasitic bronchopneumonia plays an important role in feline respiratory medicine, thus it is receiving growing attention by researchers and practitioners. In recent years, *Troglostrongylus brevior*, a lungworm usually infecting wild felids, has been recognized as an agent of the lower respiratory tract in domestic cats. In particular, as a likely consequence of a spill-over from wild reservoirs (e.g., the European wildcat), *T. brevior* infection is increasingly reported in cats from Mediterranean and Balkan countries. This parasitic nematode has an indirect life cycle, and its biology overlaps that of the better known “cat lungworm” *Aelurostrongylus abstrusus*. In fact, cases of co-infections caused by both lungworms are not infrequent in domestic cats. Knowledge on clinical features of troglostrongylosis is still incomplete. Available data indicates that clinical signs and radiographic evidence are severe especially in kittens and young cats, are non-specific and often overlap with those of other feline respiratory diseases, such as feline bronchial disease/asthma, or infectious pneumonia. These characteristics make a definitive diagnosis of troglostrongylosis challenging, this disease requires a timely ancillary therapy and an appropriate anthelminthic treatment. As feline troglostrongylosis is an emerging parasitic disease of domestic cats, it should be included in differential diagnosis for lower respiratory tract disease in cats from regions where this parasite is present but also where it is unexpected. This article reviews current knowledge on the pathogenic role of *T. brevior* in domestic cats and resulting respiratory illness, with a special focus on clinical aspects, diagnosis, and management of the disease.

## Introduction

Several parasitic nematodes cause infection of the respiratory tract in domestic cats, *Aelurostrongylus abstrusus* (Strongylida, Angiostrongylidae) being the most common and significant worldwide (1).

Metastrongyloid lungworms ranked within the genus *Troglostrongylus*, encompassing *Troglostrongylus brevior, Troglostrongylus subcrenatus, Troglostrongylus troglostrongylus*, and *Troglostrongylus wilsoni* species, have been considered affiliated only to wild felids for quite a long time (2–6). However, in recent years, the interest of the scientific community on *T. brevior* has grown due to spill-over from wild reservoirs, and recognized ability to cause infection and lower respiratory tract disease in domestic cats (7).

*Troglostrongylus brevior* was first described in the last century in two species of wild felids from Palestine (2). It was then found in central Italy in one European wildcat (*Felis silvestris silvestris*) and in a felid that the author did not clarify whether it was a feral cat or a wildcat (8). No other records of *T. brevior* were published in peer-reviewed literature until 2010, when this lungworm was described in two domestic cats living in the Ibiza Island (Spain) (9). Since then, an increasing number of reports of *T. brevior* infections in domestic cats have appeared in literature.

## Life cycle and epizootiology

Adult worms of *T. brevior* are sexually dimorphic (i.e., male: 6.6–7.2 mm in length and 0.2–0.23 mm in width; female 9.6–16.8 mm in length and 0.26–0.40 mm in width) and inhabit the bronchi and bronchioles of infected hosts. Females lay eggs hatching first-stage larvae (L1) within the respiratory tract of definitive hosts and a prepatent period of 28 days was observed in an experimentally infected kitten (2).

*Troglostrongylus brevior*, as other metastrongyloid parasites, needs a mollusc intermediate host to further develop into the infective third-stage larva (L3), and experimental studies show that several slugs and snails, such as *Chondrula septemdentata, Helicella barbesiana, Helicella ustalis, Limax flavus, Monaca syriaca, Retinella nitellina, Theba pisana*, and *Helix aspersa* may act as intermediate hosts (2, 10).

Paratenic hosts, such as rodents, birds, reptiles, and amphibians, play a pivotal role in the biology and epizootiology of the cat lungworm *A. abstrusus* (11), however their real significance in the biology of *T. brevior* is not yet well-understood. A study showed that a mouse was able to encyst L3 in experimental conditions, but further evidence of development of *T. brevior* in paratenic hosts is still lacking (2). As cats usually do not prey terrestrial molluscs because of their emetic effect, a crucial role of paratenic host in the transmission of *T. brevior* is more than plausible. Furthermore, a possible vertical transmission of the parasite has been suggested as an alternative route of transmission (12, 13).

For a long time, nematodes ranked within the *Troglostrongylus* genus have been regarded as a parasite of wild felids, such as the leopard cat (*Prionailurus bengalensis*) (14), bobcat (*Lynx rufus*) (3, 6), Canada lynx (*Felis canadensis*) (5), Eurasian lynx (*Lynx lynx*) (15), leopard (*Panthera pardus*) (16), tiger (*Panthera tigris*) (17), jungle cat (*Felis chaus*) (2), European, and Ethiopian wildcats (*Felis sivestris silvestris* and *Felis ocreata*) (2, 18, 19). In domestic cats, after the first report (9), the infection, was reported more and more in cats from Sicily, Sardinia, Crete, Skopelos, Mikonos, and Cyprus Islands (12, 20–24), peninsular and northern Italy (12, 13, 25–33), continental Greece (22, 33), Spain and Bulgaria (34). Recent surveys have shown a prevalence of *T. brevior second* only to the “cat lungworm” *A. abstrusus* (34), with a prevalence of 14% in cats from Central Italy (13), 5 and 5.6% in Cyprus and Greece, respectively (23), and 1.2% in the Sardinia Island (24). These findings indicate that troglostrongylosis is an up-and-coming disease of the domestic cat across Southern European countries.

The origin of this spread among the population of domestic cats is likely related to a spill-over effect, that occurs when a reservoir population, affected by high pathogen prevalence, interacts with a susceptible one (35). *Troglostrongylus brevior* has been isolated from domestic cats living in regions where populations of wild cats live in sympatry and are infected by this lungworm with high percentages (36). Furthermore, domestic and wildcats share the same haplotypes in some regions (32), further proving the host switching of *T. brevior* from wild to domestic cats. However, feline troglostrongylosis has been reported also in regions where the natural host responsible for the spread of the infection (i.e., *F. s. silvestris*) is not documented (9, 33). It is possible that movements of paratenic hosts may have introduced infective stages of *T. brevior* in these areas as well as that cats traveling with their owners from endemic areas to previously free regions could have imported this lungworm (33). Finally, it has been suggested that, in given regions, such as small islands, this parasite has adapted to domestic cats in pre-historical times, before the present coastal arrangement appeared, when the wildcat or a common ancestor was likely present (33).

## History and clinical signs

*Troglostrongylus brevior* infection occurs mostly in cats younger than 1 year (23, 34, 36), often causing severe and life-threatening lower respiratory tract disease (12, 20, 27, 31, 37). Nonetheless, subclinically infected animals and cases of mild clinical signs have been reported, both in young and adult cats (12, 27, 31). The infection should be suspected in young cats with a history of outdoor life (9, 12, 13, 20–34).

The most common signs in cats with troglostrongylosis are cough, dyspnea and tachypnea (12, 20, 22, 30, 31, 37). However, clinical signs of lower respiratory tract disease are a common presenting complaint in cats of all ages, and such manifestations frequently overlap with other respiratory disorders (31). Furthermore, other not disease-specific signs, such as anorexia/hyporexia, hyperthermia/hypothermia, dehydration, poor body conditions, and lethargy are reported in feline troglostrongylosis (22, 31). Ocular and/or nasal discharge and sneezing are typical signs of feline upper respiratory tract disease (URTD), and these clinical signs have been reported in lungworm and *T. brevior* infections as well (20, 31), even in the absence of the most common pathogens involved in URTD (31). Despite this evidence, the mechanism leading to upper respiratory signs in cats affected by lungworms is still unclear, even though a dislocation of bronchial secretions in the nasal cavity has been proposed (31). However, the concurring presence of infectious agents of URTD and lungworms, especially in young and multi-housed cats, is also likely.

Lung auscultation can reveal increased vesicular breath sounds, usually bilaterally, and wheezing (31). The presence of a right systolic heart murmur has been reported in a 4-month old kitten, with a pulmonary hypertension associated with *T. brevior* infection, suggesting an important role of the examination of the cardiocirculatory system, especially in those cats with severe respiratory signs, in order to investigate the onset of any possible cardiac complications (30).

## Diagnosis

### Hematobiochemical analysis

In general, laboratory work in cats with respiratory disease can help to narrow differential diagnosis, to address further tests, to assess co-morbidity, and to give a prognosis. Unfortunately, to date, very few data are available on CBC, serum biochemistry, serum protein electrophoresis, and urinalysis in feline troglostrongylosis and they derive from case reports and case series only (20, 31).

The CBC was found within normal limits in the majority of cats affected by *T. brevior* and serum biochemistry examination of these animals was unremarkable (20, 31). Peripheral eosinophilia is occasionally observed in cats with parasitic lung disease, but in a recent study, this finding was not observed in 10 cats affected by *T. brevior*, in both single and mixed lungworm infections (31). Leukocytosis is a common finding in feline bronchial disease and it was observed in two 3-month old female kittens with a mono-specific infection caused by *T. brevior* (20, 31). In one of these cats mild anemia, neutrophilia, and monocytosis, likely as a result of the chronic inflammation, were also observed (31).

### Imaging

Thoracic radiography should be the first assessment in patients with a suspect of lower airway and pulmonary parenchyma disorders. The most common abnormalities detected on chest radiographs in naturally (31, 38, 39) or experimentally (40) infected cats with lungworm infection are bronchial, nodular, and unstructured interstitial pattern with a multifocal distribution, evolving into a generalized alveolar pattern in severe cases. However, these data relate to *A. abstrusus* or mixed lungworm infections, and information on imaging features of troglostrogylosis is scant.

**Figure 1:**
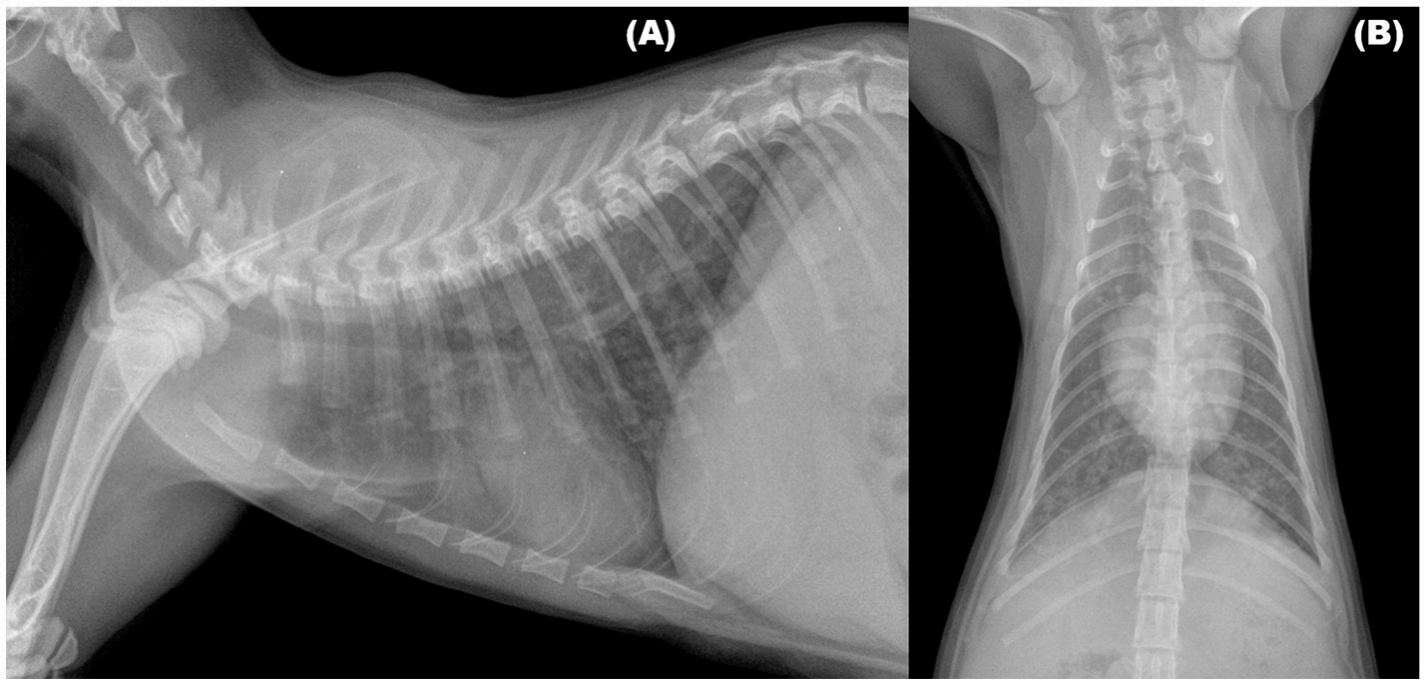
Right lateral **(A)** and Ventrodorsal **(B)** views of the thorax of a 3-month old kitten infected by *Troglostrongylus brevior*. An unstructured interstitial pattern with severe bronchial thickening were visible through the lung lobes.

Radiographic signs of troglostrongylosis range from mild to severe, and, non-specific patterns, as interstitial, bronchial, and alveolar, either alone or in association, have been reported (Figure 1). Other abnormalities, such as nodular lesions or vascular patterns, were observed in cats with poly-specific infections sustained by *A. abstrusus* and *T. brevior* (30, 31). Interestingly, as already observed in feline bronchopneumonia, including aelurostrongylosis (38, 41, 42), in two kittens with troglostrongylosis a disagreement, between clinical and radiographic signs, was reported. In particular, interstitial changes were observed in an asymptomatic kitten, while a bronchointerstitial involvement was highlighted in a kitten showing only signs of upper respiratory tract disease (i.e., oculo-nasal discharge and sneezing), confirming that radiographic changes may be evident before the onset of clinical signs in feline lungworm disease (31, 38).

### Copromicroscopic examination and biomolecular assays

For the reasons above a clinical diagnosis is virtually impossible, and clinical, laboratory, and radiographic signs allow only a suspicion of a lower respiratory tract disorder. Thus, a definitive diagnosis of troglostrongylosis can be achieved with observation of parasites or their DNA in biological samples (1).

To date there is no evidence of tracheal wash or BAL cytology as tools for diagnosing troglostrongylosis, but the low sensitivity observed for aelurostrongylosis does not justify the use of this invasive procedure, especially in severe cases (43).

Copromicroscopic examination is a simple, cheap and reliable in-house test, and should always be considered as a first step in the diagnostic work-up for feline lower airway diseases, especially in at-risk subjects (31). As for other metastrongylid parasites, the Baermann test is the gold standard to diagnose feline troglostrongylosis (1). However, this technique has some limitations; first of all, it may be necessary to leave the specimen set up overnight and repeated examinations are necessary to enhance its sensitivity, thus, in some cases, it may take several days. Furthermore, a discrimination between L1s of *A. abstrusus*, as well as other metastrongyilid larvae (e.g., *Oslerus rostratus*), and L1s of *T. brevior* is difficult and requires a careful examination and specific skills (1) (Table 1).

**Table 1:** Key features for identification of the first larval stage of Metastrongyloidea in faces of cats.

**Nematode**	**Morphological features**	**Length (μm)**	**References**
*Aelurostrongylus abstrusus*	Head: rounded, terminal oral opening Tail: kinked (S-shaped), knob-like or small finger-like projections at tip of cuticular spines	360–415^*^	(1)
*Troglostrongylus brevior*	Head: pointed, subterminal (dorsal) oral opening Tail: gradually tapered to the extremity, deep dorsal incisure and shallower ventral incisure (slight individual variation)	300–357^*^	(1)
*Troglostrongylus subcrenatus*	Head: pointed, subterminal (dorsal) oral opening Tail: gradually tapered to the extremity, deep dorsal incisure and shallower ventral incisure	269–300	(20)
*Oslerus rostratus*	Head: oral opening central to the head and surrounded by a cuticular ring with dorsal and ventral prominences Tail: deep incisure in the ventral side with a cuticular spine (slight individual variation)	216–412	(34, 44)
Angiostrongylus chabaudi^**^	Head: rounded, terminal oral opening Tail: kinked (S-shaped), subterminal spine separated by a notch, small ventral incisure	362–400	(45)

* *A variation in length of first stage larvae has been reported for Aelurostrongylus abstrusus (minimum 210 μm, maximum 495 μm) and Troglostrongylus brevior (minimum 203 μm, maximum 382 μm)*.

** *larvae collected from a wildcat (Felis silvestris)*.

Biomolecular assays make it possible to overcome these limitations and diagnostic methods have been validated for the genetic identification of *T. brevior* (1). A species-specific nested PCR based on differential markers within the ribosomal (rdNA) internal transcribed spacer 2 region (ITS2) proved effective in identifying *T. brevior* in different biological samples from infected cats (e.g., faces and pharyngeal swabs) (27, 46); furthermore, a duplex PCR assay on markers within ITS2 was developed to discriminate *A. abstrusus* and *T. brevior* L1s in a single cat with a mixed infection (26). Finally, a triplex semi-nested PCR specific for the ITS2 region has been validated for the simultaneous discrimination of *A. abstrusus, T. brevior*, and *Angiostrongylus chabaudi* (47).

## Therapy

### Supportive care

As mentioned above, dyspnea is a common presentation of feline troglostrongylosis, and in kittens with severe respiratory signs death can occur despite a prompt administration of anthelmintic compounds (20, 27, 31, 46). Thus, in an effort to improve outcomes, supportive care measures are of key importance.

Because stress and handling can exacerbate respiratory signs, care must be taken to not overly stress the cat, and initial treatment may include cage rest and supplemental oxygen. Since these patients, especially kittens, have the ability to decompensate quickly and stress increases the respiratory rate and demand for oxygen, anxiolysis is recommended and, for instance, butorphanol may be given for both anxiolytic and antitussive effects. Furthermore, the administration of short-acting corticosteroids, such dexamethasone sodium phosphate can help to reduce bronchial inflammation (48–50). A fluid therapy should be considered in those subject with poor clinical conditions (48–50). The role of secondary bacterial infections in lungworm disease is still unknown, and the administration of antibiotics in cats affected by lungworms is questionable and should be evaluated on a case-by-case basis.

### Etiological therapy and prevention

At present only spot-on eprinomectin is licensed for treating feline troglostrongylosis, although different compounds have shown promising performance in cats naturally infected with *T. brevior*.

A single dose of spot-on eprinomectin assured parasitological negativization and clinical recovery in cats with monospecific infection by *T. brevior* and in cats with *T. brevior* and *A. abstrusus* poli-specific infection (28, 34).

Spot-on moxidectin was proven effective against *T. brevior*, in both single and mixed lungworm infections. In particular, a single topical application of moxidectin was effective in assuring parasitological negativization in two kittens with troglostrongylosis. In three cats co-infected by *A. abstrusus* and *T. brevior*, two or three moxidectin administrations, 2 weeks apart, were necessary in order to achieve the parasitological negativization. In all such cases, a full clinical recovery was observed in 2–6 weeks after the first administration (31).

No data are currently available on the efficacy of emodepside in treating troglostrongylosis, nevertheless one administration of a spot-on emodepside was effective in assuring clinical recovery and reducing larval shedding of *T. brevior* in kittens with a mixed infection, either with *A. abstrusus* or *C. aerophila* (27, 31). In a further kitten, infected by *T. brevior* in association with *A. abstrusus*, parasitological negativization and disappearance of clinical signs was recorded after two administrations of emodepside, once every 2 weeks (27).

Clinical signs and larval shedding disappeared after a single oral administration of milbemycin oxime (2 mg/kg body weight) in a kitten with troglostrongylosis and in a kitten infected by *T. brevior* together with *A. abstrusus* (31).

To date, these compounds have proved their efficacy with an intermediate-weak degree of evidence (i.e., EBM level IV) (50, 51) and large-scale studies are necessary to ultimately investigate their efficacy and safety in cats with troglostrongylosis. Nevertheless, different therapeutic options may be used and the anthelminthic compound should be selected based on (i) age and weight of the cats, (ii) indole of the cats, and (iii) owner compliance.

The majority of cats affected by troglostrongylosis are young (i.e., < 2–6 months) and all of the above-mentioned products are licensed for use in kittens also starting from 6 to 8 weeks of age and a body weight of 0.5–1 kg (52–55). In general, oral products can be problematical to administer especially in indocile and feral cats while easy-to-apply topical parasiticides are a suitable choice for treating cat lungworms, because of ease of administration, especially when multiple dosing is required (56, 57).

Cats totally or partially living outdoors have a high risk to be infected by *T. brevior*, and virtually avoiding external access and subsequently reducing chances of predation is at present the only preventive measure (50). However, this measure is impracticable especially in multi-housed and stray cats. No data are available on chemoprevention of troglostrongylosis, though the potential efficacy of different formulations in the prevention of closely-related metastrongyloid infections was observed. For instance, the formulation containing eprinomectin was able to prevent the infection in experimentally infected cats (58), and spot-on moxidectin is efficacious in preventing the infection by *Angiostrongylus vasorum* in dogs (59, 60). Moxidectin levels are detectable for weeks after treatment (61), and regular administrations of topical moxidectin can induce elevated and sustained steady-state plasma concentrations (52). For instance, in cats, three to five treatments allow to reach a steady-state concentration effective against hookwoorms and *Dirofilaria immitis* infections (62, 63).

## Prognosis and outcome

A correct diagnosis and an effective anthelminthic therapy often assure a full remission of signs and parasitological negativization within 2–6 weeks (31). Cats receiving outpatient care should be evaluated for clinical condition every week and larval shedding monitored every 2 weeks until clinical and parasitological recovery (31).

Cats, especially young ones, that generally show severe clinical presentation, require hospitalization. These animals can decompensate quickly and may die after an acute respiratory distress, even with intensive therapy and anthelmintic treatment (20, 30, 31). Thus, in these animals the prognosis is guarded to poor, and the owners should be informed about long-term complications, even rare ones, such as pulmonary hypertension (30).

## Conclusions

Feline troglostrongylosis is an emergent disease thus, also considering its clinical severity, feline practitioners should be aware of *T. brevior* in regions where this parasite is present as well as where it is unexpected.

Feline troglostrongylosis should be considered in differential diagnosis for lower respiratory airway disease in cats with outdoor access or in free-roaming animals and copromicroscopic examination should always be considered as a first step in the diagnostic work-up for any cat with respiratory signs. Furthermore, many cats with lower airway diseases may have a subclinical infection and *T. brevior*, as also other lungworms, can cause radiographic changes even in absence of overt clinical signs (31), indicating that lung damage can be present regardless of the clinical presentation.

## Author contributions

All authors listed have made a substantial, direct and intellectual contribution to the work, and approved it for publication.

### Conflict of interest statement

The authors declare that the research was conducted in the absence of any commercial or financial relationships that could be construed as a potential conflict of interest.
